# Adenosine signaling: Optimal target for gastric cancer immunotherapy

**DOI:** 10.3389/fimmu.2022.1027838

**Published:** 2022-09-16

**Authors:** Junqing Wang, Linyong Du, Xiangjian Chen

**Affiliations:** ^1^School of the 1St Clinical Medical Sciences, Wenzhou Medical University, Wenzhou, China; ^2^Key Laboratory of Laboratory Medicine, Ministry of Education of China, School of Laboratory Medicine and Life Science, Wenzhou Medical University, Wenzhou, China

**Keywords:** gastric cancer, CD39, CD73, adenosine, immunotherapy

## Abstract

Gastric cancer (GC) is one of the most common malignancy and leading cause of cancer-related deaths worldwide. Due to asymptomatic or only nonspecific early symptoms, GC patients are usually in the advanced stage at first diagnosis and miss the best opportunity of treatment. Immunotherapies, especially immune checkpoint inhibitors (ICIs), have dramatically changed the landscape of available treatment options for advanced-stage cancer patients. However, with regards to existing ICIs, the clinical benefit of monotherapy for advanced gastric cancer (AGC) is quite limited. Therefore, it is urgent to explore an optimal target for the treatment of GC. In this review, we summarize the expression profiles and prognostic value of 20 common immune checkpoint-related genes in GC from Gene Expression Profiling Interactive Analysis (GEPIA) database, and then find that the adenosinergic pathway plays an indispensable role in the occurrence and development of GC. Moreover, we discuss the pathophysiological function of adenosinergic pathway in cancers. The accumulation of extracellular adenosine inhibits the normal function of immune effector cells and facilitate the effect of immunosuppressive cells to foster GC cells proliferation and migration. Finally, we provide insights into potential clinical application of adenosinergic-targeting therapies for GC patients.

## Introduction

Gastric cancer (GC) is a major source of global cancer mortality with limited treatment options and poor patient survival. It is the fourth most commonly occurring cancer in men and the seventh in women (1). For patients with early gastric cancer (EGC) and low risk of lymph node metastasis, endoscopic submucosal dissection (ESD) or radical surgical resection alone is potentially curative (2, 3). Unfortunately, due to no apparent symptom or only indigestion-like clinical manifestations, such as inappetence, gastroesophageal reflux, and belching, patients with EGC often miss the best treatment opportunity because of negligence (2). Although endoscopic screening significantly increases the detection of EGC and improves prognosis (4). Skill among endoscopists varies greatly, and numerous patients are still missed for various reasons (5). As the disease progresses, hemorrhage, perforation, obstruction, cachexia, and other symptoms of advanced cancer gradually appear. GC is already in the advanced stage once detected in patients, which has a poor ending due to ineffective therapies and multiple resistance (6). Therefore, accurately diagnosing EGC and effectively treating advanced gastric cancer (AGC) patients who have lost the chance of radical surgical resection are two serious health problems all over the world.

For the patients who are suffering from GC, the treatments are mainly surgical excision, chemotherapy, targeted therapy, immunotherapy, and other comprehensive strategies (7). Among them, radical gastrectomy with D2 lymphadenectomy, with or without neoadjuvant therapy, is the only potentially curative treatment option (8). However, increasing numbers of studies have shown that surgery cannot benefit patients with unresectable AGC and post-operative complication is a negative predictor of long-term survival outcomes for them (9). Systemic chemotherapy with multiple drug regimens is the main therapy choice to further prolong the survival of post- or non-operative AGC patients (10). Despite relevant progress, the impact of chemotherapy on AGC patients’ survival is still unsatisfactory, especially patients with multiple distant metastases (1). Additionally, as an emerging, attractive, and effective treatment, targeted therapy has shown promising effects in a part of GC patients, even if the beneficiary degree not definite (11). As the most common target in GC, the frequency of human epidermal growth factor receptor 2 (HER2) overexpression ranges from 4.4% to 53.4%, with a mean of 17.9% (12). Coupled with drug resistance developed during treatment, management of AGC patients by targeted therapy remains a challenge. Despite new therapeutic options, AGC remains associated with a poor prognosis compared with other cancers, on account of inactive immunogenicity and vast heterogeneity represent a barrier to disease management (13, 14).

Immunotherapies, especially immune checkpoint inhibitors (ICIs) and chimeric antigen receptor-modified T (CAR T) cell therapies, have been used continuously for decades, as lifesaving procedures for millions of patients with hematological malignancy (15). As the most extensively used ICIs at present, checkpoint inhibitor-based immunotherapies that target the cytotoxic T lymphocyte-associated antigen 4 (CTLA-4) and the programmed cell death receptor 1/programmed cell death ligand 1 (PD-1/PD-L1) pathway have achieved impressive success in the treatment of different cancer types (16). Nevertheless, there still exists various challenges that have severely limited the clinical application of immunotherapies in AGC, for instance, the ineffectiveness and serious side effects (6). For AGC patients, anti-CTLA-4 and anti-PD-1/PD-L1 monoclonal antibodies cannot acquire satisfactory curative effect without the assistance of other cancer treatments (17–20). Some clinical trials have shown positive effects on overall response and disease control in combination with ICIs and other therapies, yet responses are slight and heterogeneous (17). Therefore, it is urgent to explore a more effective immunotherapy method to prolong the survival of AGC patients.

In this review, we find that CD73 is the most important immune checkpoint affecting the prognosis of GC patients by analyzing the Gene Expression Profiling Interactive Analysis (GEPIA) database. In addition, we also describe the mechanism of CD39-CD73-adenosine signaling pathway in immune regulation of cancers and discuss its role in the occurrence and development of GC. At the end of the article, we also put forward some prospects about treating GC with the help of targeting CD39-CD73-adenosine axis.

## CD73 is an optimal target for GC immunotherapy

ICI, especially inhibition of PD-1/PD-L1 axis, is a new standard of immunotherapy in the treatment of advanced or metastatic GC and is represented in various combinations with and without other treatments within clinical trials (21). However, its curative effect is related to individual differences to a certain degree. For example, in a randomized, open-label, phase 3 trial (NCT02370498), the PD-1/PD-L1 blockade cannot significantly improve overall survival (OS) and progression-free survival (PFS) versus paclitaxel for PD-L1-positive GC (all P > 0.6) (22). In another phase 3 randomized clinical trial (NCT02494583), the PD-1/PD-L1 blockade plus chemotherapy was not superior to chemotherapy for OS (12.3 vs. 10.8 months; HR, 0.85; 95% CI, 0.62-1.17; P = 0.16) (19). Collectively, the immunotherapy of GC needs a more appropriate immune checkpoint to obtain superior efficacy.

**Figure 1:**
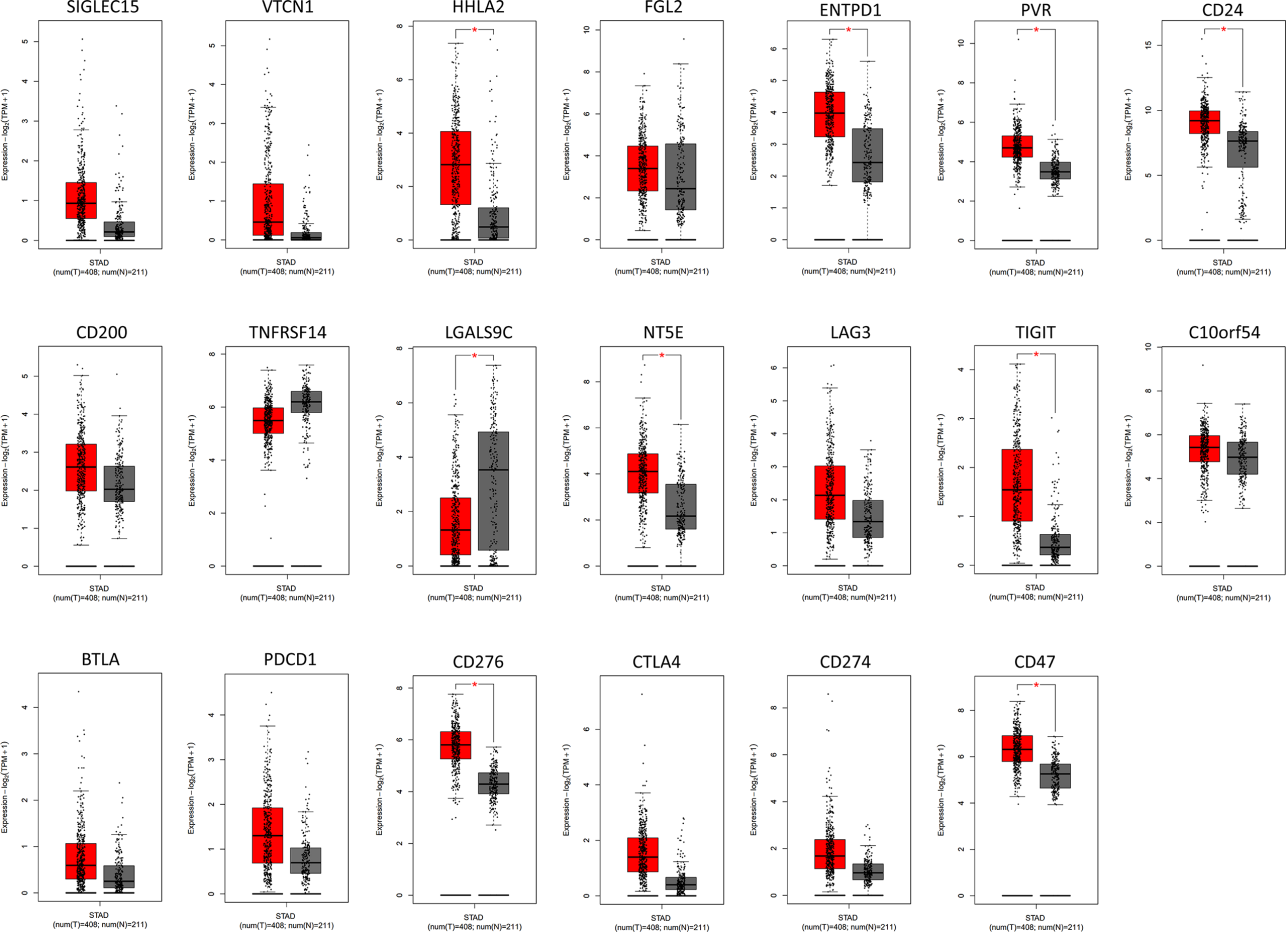
The analysis of immune checkpoint-related genes expression in GC by GEPIA database. The results revealed that 9 genes were confirmed to have significant differential expression in GC compared to the normal tissues. Among them, higher expression was observed in HHLA2, ENTPD1, PVR, CD24, NT5E, TIGIT, CD276, and CD47 and lower expression was observed in LGALS9C. Red color represents tumor tissue (n=408), and gray color represents normal tissue (n=211). STAD, stomach adenocarcinoma. * P < 0.05.

To further confirm which target plays the most indispensable role in GC, we input 20 common immune checkpoint-related genes into the GEPIA server for in-depth analysis (**Table 1**). Among them, we found that 9 genes were confirmed to have significant differential expression in GC (**Figure 1**). Moreover, the expression levels of PDCD1 (encode PD-1), CD274 (encode PD-L1), and CTLA-4 genes in GC not change compared with adjacent tissues, which was consistent with the above-mentioned treatment results.

**Table 1:** The characteristics of 20 immune checkpoint-related genes.

Gene Names	Protein Names	Subcellular Location	Normal Tissue Specificity	Cancer Types	Function	References
**SIGLEC15**	sialic acid-binding Ig-like lectin 15	plasma membrane	macrophage and/or dendritic cells of spleen and lymph nodes	lymphoma, leukemia, thyroid cancer, and renal cell cancer	TAM-associated Siglec-15 has a potent immune suppressive effect on T-cell responses	(23, 24)
**VTCN1**	V-set domain-containing T-cell activation inhibitor 1	plasma membrane	activated T- and B-cells, monocytes, and dendritic cells	breast cancer, ovarian cancer, and renal cell cancer	negatively regulates T-cell-mediated immune response by inhibiting T-cell activation, proliferation, cytokine production and development of cytotoxicity	(25, 26)
**HHLA2**	human endogenous retrovirus-H long terminal repeat-associating protein 2	plasma membrane	colon, kidney, testis, B-cells, and dendritic cells	colorectal cancer, pancreatic cancer, and gastric cancer	inhibits CD8^+^ T and NK cell function and killing	(27, 28)
**FGL2**	fibroleukin	extracellular region and exosome	cytotoxic T-cells	leukemia and lymphoma	induces CD8 ^+^ T cell apoptosis to limit T cell immunity through the inhibitory Fc receptor FcγRIIB	(29, 30)
**ENTPD1**	ectonucleoside triphosphate diphosphohydrolase 1 (CD39)	plasma membrane	activated lymphoid cells and endothelial tissues	glioma, gastric cancer, and renal cell cancer	hydrolyzes eATP and eADP into eAMP to provide raw materials for CD73	(31, 32)
**PVR**	poliovirus receptor (CD155)	cytoplasm, cell surface and extracellular space	widely expressed	esophageal carcinoma, adrenocortical carcinoma, and colon carcinoma	provides tumors with a mechanism of immunoevasion from NK cells	(33, 34)
**CD24**	signal transducer CD24	cell surface	B-cells	Breast cancer, colorectal cancer, and gastric cancer	regulates the proliferation of B-cells and prevents their terminal differentiation into antibody-forming cells	(35, 36)
**CD200**	OX-2 membrane glycoprotein	cell membrane	widely expressed	pheochromocytoma, paraganglioma and renal cell cancer	inhibits T-cell proliferation	(37, 38)
**TNFRSF14**	tumor necrosis factor receptor superfamily member 14 (CD270)	cell membrane	lung, spleen, and thymus	melanoma, lymphoma, and lung cancer	synergistically inhibits the function of lymphocytes with BTLA	(39, 40)
**LGALS9C**	galectin-9C	cytosol and nucleus	widely expressed	head and neck squamous cell carcinoma, and colorectal cancer	interacts with multiple molecules to regulate immune cells proliferation and death	(41, 42)
**NT5E**	5’-nucleotidase (CD73)	cell membrane	activated lymphoid cells and endothelial tissues	thyroid cancer, gastric cancer, sarcoma, and glioma	hydrolyzes eAMP into immunosuppressive adenosine	(43, 44)
**LAG3**	lymphocyte activation gene 3 protein (CD223)	cell membrane and extracellular region	activated T-cells and NK cells	leukemia and testicular germ cell tumors	negatively regulates the proliferation, activation, effector function and homeostasis of both CD8^+^ and CD4^+^ T-cells	(45, 46)
**TIGIT**	T-cell immunoreceptor with immunoglobulin and ITIM domains	cell membrane	T-cells and NK cells	leukemia and lung adenocarcinoma	suppresses T-cell activation by promoting the generation of mature immunoregulatory dendritic cells	(47, 48)
**C10orf54**	V-type immunoglobulin domain-containing suppressor of T-cell activation (VISTA)	cell membrane	placenta, spleen, plasma blood leukocytes, and lung	leukemia and pancreatic cancer	immunoregulatory receptor which inhibits the T-cell response	(49, 50)
**BTLA**	B- and T-lymphocyte attenuator (CD272)	cell membrane	lymph node	lymphoma and leukemia	inhibitory receptor on lymphocytes that negatively regulates antigen receptor signaling	(51, 52)
**PDCD1**	programmed cell death protein 1 (PD-1)	cell membrane	induced at programmed cell death	lymphoma, melanoma, and lung cancer	plays a critical role in induction and maintenance of immune tolerance	(53, 54)
**CD276**	CD276 antigen	cell membrane	peripheral blood lymphocytes or granulocytes	sarcoma, glioma, lung cancer, and prostate cancer	inhibits T-cell-mediated immune response and NK cell-mediated lysis	(55, 56)
**CTLA4**	cytotoxic T-lymphocyte protein 4	cell membrane, Golgi apparatus, cytoplasm	widely expressed	lymphoma, leukemia melanoma, and lung cancer	inhibitory receptor acting as a major negative regulator of T-cell responses	(57, 58)
**CD274**	programmed cell death 1 ligand 1 (PD-L1)	cell membrane, nucleoplasm, and extracellular exosome	lung, heart, placenta, and kidney	lymphoma, melanoma, and lung cancer	as a ligand for the inhibitory receptor PD-1, modulates the activation threshold of T-cells and limits T-cell effector response	(53, 54)
**CD47**	leukocyte surface antigen CD47	cell surface and extracellular exosome	widely expressed	leukemia, ovarian cancer, lung cancer, and pancreatic cancer	prevents maturation of immature dendritic cells and inhibits cytokine production by mature dendritic cells	(59, 60)

TAM, tumor-associated macrophage; NK, natural killer cell; eAMP, extracellular adenosine monophosphate; eADP, extracellular adenosine diphosphate; eATP, extracellular adenosine triphosphate; ITIM, immunoreceptor tyrosine-based inhibitory motif.

**Figure 2:**
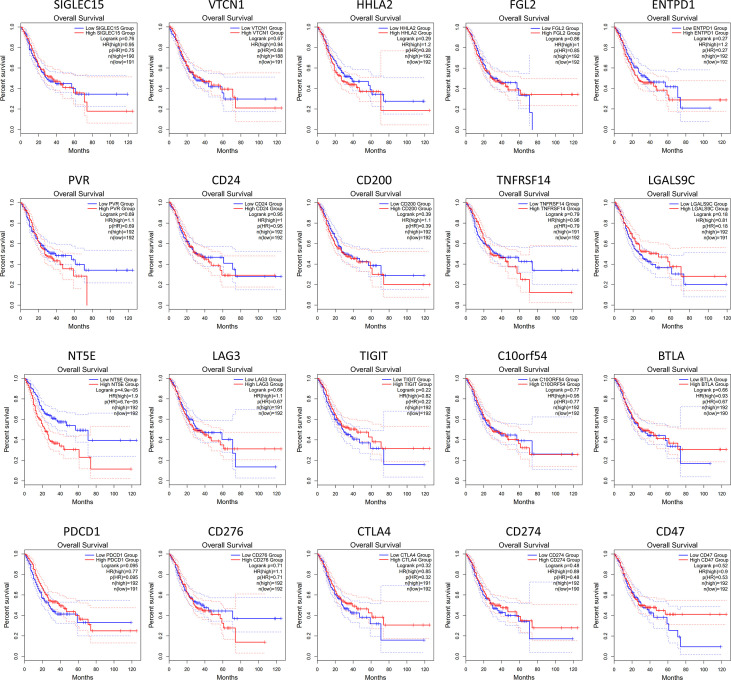
Kaplan-Meier survival curves comparing the high and low expression of immune checkpoint-related genes in GC by GEPIA database. The results showed that only the high expression of NT5E (encode CD73) was correlated with poor prognosis of GC patients (p<0.05). The red line indicates the high expression group of genes (n=192) and the blue line represents the low expression group of genes (n=191).

Furthermore, we investigated whether the expression of various immune checkpoint-related genes was correlated with prognosis in GC patients (**Figure 2**). The results of GEPIA analysis showed that only the high expression of NT5E (encode CD73) is more likely to encounter GC patients death earlier and shorten survival time (p<0.05). Additionally, with the help of immunohistochemistry, single-sample gene set enrichment analysis and flow cytometry, extensive related studies have reported that CD73 expression is upregulated in GC which is proved to be an independent adverse prognosticator for the patients (61–63).

Ecto-5’-nucleotidase (NT5E), also known as CD73, is a cytomembrane protein linked to the cell membrane *via* a glycosylphosphatidylinositol (GPI) anchor that regulates the conversion of extracellular adenosine monophosphate (eAMP) to adenosine contributing to immunosuppression (64). CD39, also termed ectonucleoside triphosphate diphosphohydrolase-1 (ENTPD1), catalyzes the hydrolysis of extracellular adenosine triphosphate (eATP) and adenosine diphosphate (eADP) into eAMP to provide raw materials for CD73 (65). As the end product of CD39-CD73 axis, adenosine mediates immunosuppression within the tumor microenvironment (TME) through triggering adenosine receptors on the membrane surface, including A1R (encoded by ADORA1), A2AR (encoded by ADORA2A), A2BR (encoded by ADORA2B), and A3R (encoded by ADORA3) (66).

**Figure 3:**
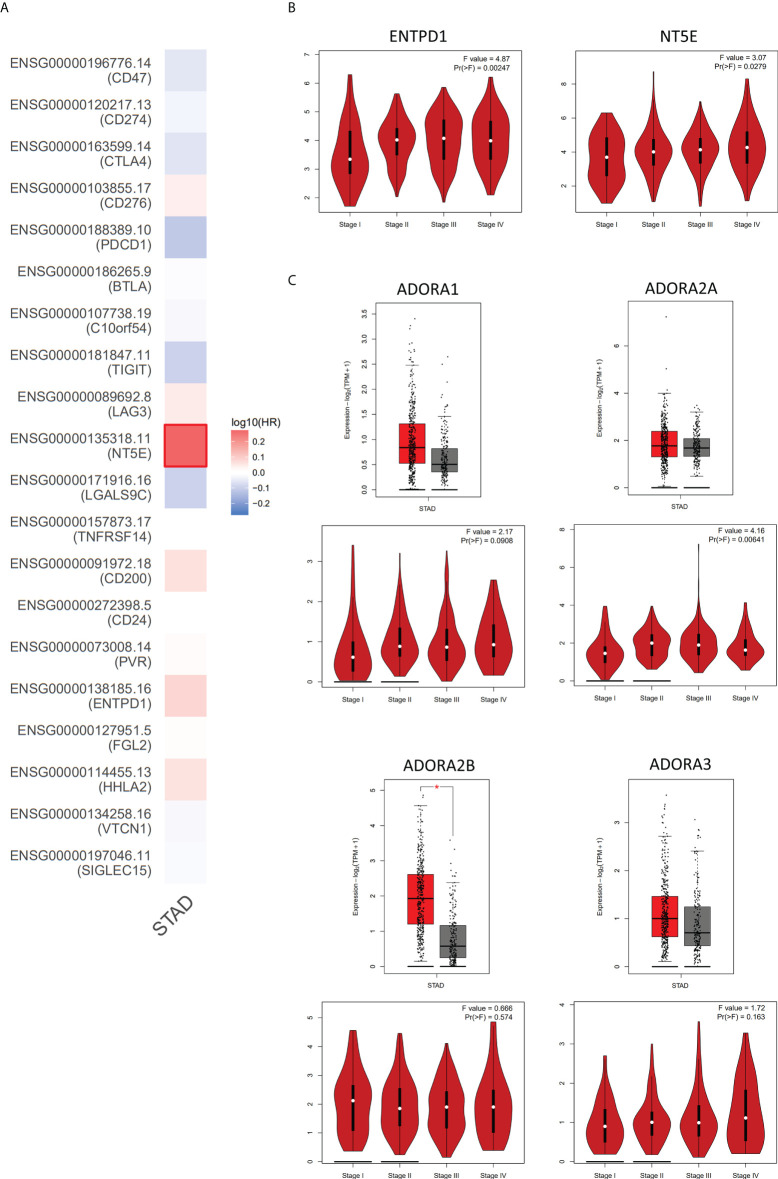
The analysis of adenosinergic pathway-related genes expression in GC by GEPIA database. **(A)** The risk assessment of 20 common immune checkpoint-related genes affecting the prognosis of GC patients. By comparing the survival contribution of multiple genes *via* Mantel-Cox test, we found that NT5E (encode CD73) showed the most obvious detrimental role in GC patients (n=383). **(B)** The expression levels of ENTPD1 and NT5E in different tumor stages of GC. With the progression of GC, the expression of ENTPD1 and NT5E also increased. **(C)** The expression levels of adenosine receptors in GC patients. The analysis showed that only ADORA2B expression (encode A2BR) increased in GC compared to the normal tissues and only ADORA2A (encode A2AR) was positively correlated with the progression of GC. Red color represents tumor tissue (n=408), and gray color represents normal tissue (n=211). STAD, stomach adenocarcinoma; HR, hazard ratio. * P < 0.05.

Based on these, we analyzed the associations between 20 common immune checkpoint-related genes and survival contribution in GC by GEPIA database. In general, compared with other immune checkpoints, CD73 showed the most obvious detrimental role in GC patients (**Figure 3A**). In addition, according to the analysis of corresponding genes expression and the TNM stage, we also found that the expression of CD39 and CD73 was higher in GC patients with clinic stage II, stage III, or stage IV than that in stage I, which revealed that these upregulated genes might be associated with tumor progression positively (**Figure 3B**). However, the role of adenosine receptors in GC patients still needs to be further evaluated (**Figure 3C**).

Taken together, the CD39-CD73-adenosine signaling pathway, as the most important immune checkpoint in GC, mediates the immunosuppressive mechanism by which tumors escape immunosurveillance and impede anti-tumor immunity within the TME. Thereinto, CD73 is an optimal target for the immunotherapy of GC.

## The CD39-CD73-adenosine signaling pathway in cancers

### eATP and immune response

**Figure 4:**
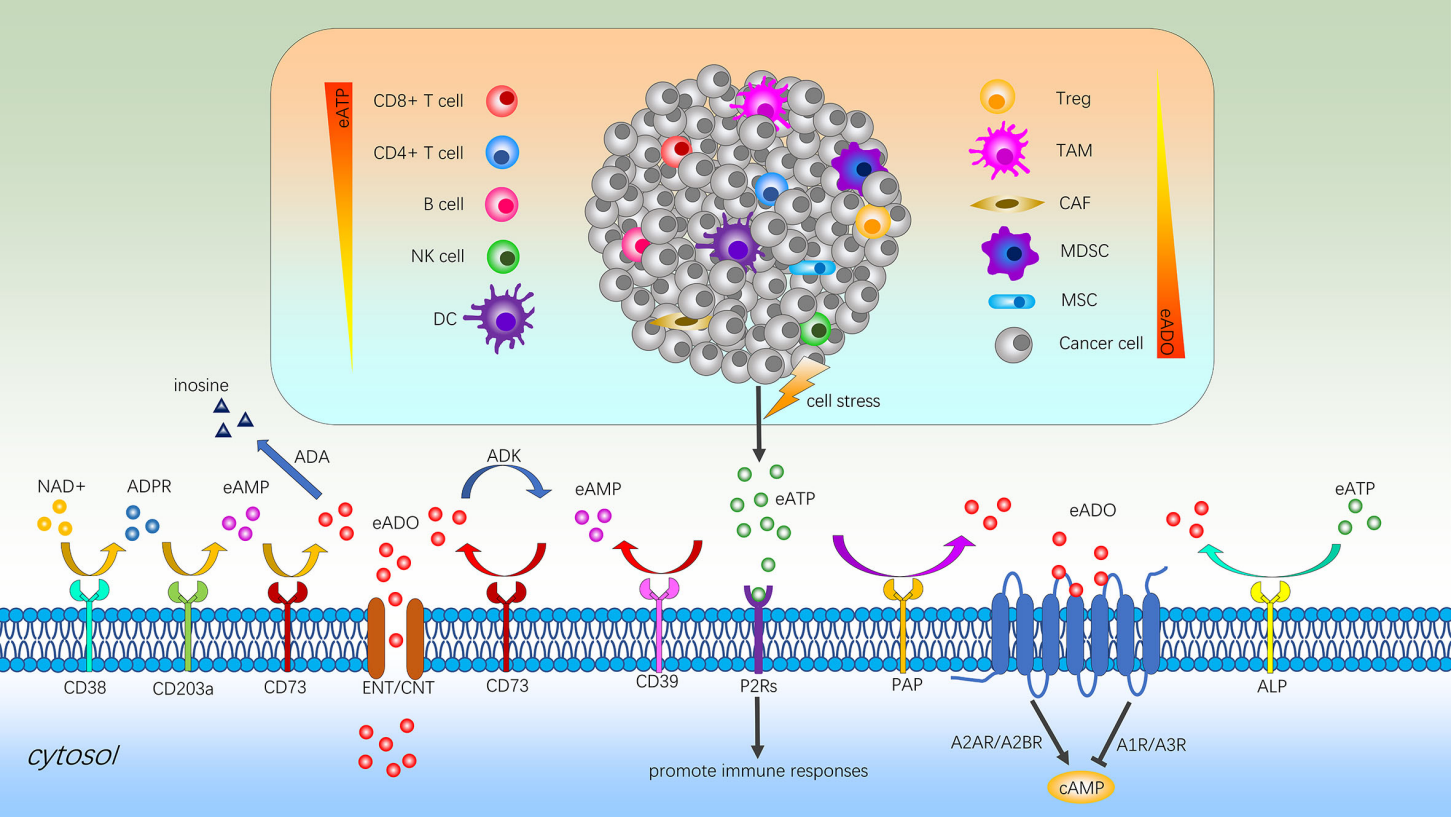
Immune regulation of adenosine signaling in the TME. Cell stress promotes eATP production and contributes to chronic inflammation *via* P2Rs. Within the TME, accumulated eATP can be degraded to ADO by the sequential action of the ectonucleotidases CD39 and CD73 or other alternative pathways such as ALP or PAP-mediated process. In addition, the sequential catabolism of NAD^+^ by CD38, CD203a and CD73 also can generate ADO and the high concentration of intracellular ADO can be transported outside the cell *via* ENTs or CNTs to maintain balance. The bioavailability of extracellular ADO is regulated by adenosine-converting enzymes such as ADK and ADA, which converts ADO into AMP and inosine respectively. High concentrations of ADO binding to adenosine receptors to inhibit the activation of immune cells and stimulate immunosuppressive cells to promote the immune escape of cancers. eATP, extracellular adenosine triphosphate; eAMP, extracellular adenosine monophosphate; NK cell, natural killer cell; DC, dendritic cell; Treg, regulatory T cell; TAM, tumor-associated macrophage; CAF, cancer associated fibroblast; MDSC, myeloid-derived suppressor cell; MSC, mesenchymal stromal cell; ADO, adenosine; NAD^+^, nicotinamide adenine dinucleotide; ADPR, adenosine diphosphate ribose; ADA, adenosine deaminase; ADK, adenosine kinase; ENT, equilibrative nucleoside transporter; CNT, concentrative nucleoside transporter; P2Rs, P2 purinergic receptors; PAP, prostatic acid phosphatase; ALP, alkaline phosphatase; cAMP, cyclic adenosine monophosphate.

Under normal circumstances, ATP is almost exclusively present inside cells as the main energy currency, participating in virtually all biological processes (67). eATP, as an extracellular messenger, is set by both passive and active release mechanisms and degradation processes (68, 69). Measurement of eATP levels in different biological context reveals that healthy tissues present very low levels (10–100 nanomoles per liter) of this nucleotide in the pericellular space, while in sites of tissue damage, inflammation, hypoxia, ischemia or TME it can reach high levels (100–500 micromoles per liter) to promote inflammatory responses (**Figure 4**) (70, 71).

There are two families of P2 purinergic receptors (P2Rs) for eATP: ATP-gated ion channels known as P2X receptors (P2X1-7) and G protein–coupled P2Y receptors (P2Y1, P2Y2, P2Y4, P2Y6, P2Y11b, P2Y12, P2Y13, P2Y14c) (69). Among them, the P2X7 receptor (P2X7R), as the most structurally and functionally distinct P2R subtype, appears to be a main player in host-tumor cell interactions because of involvement in apoptotic, inflammatory, and tumor progression pathways (72, 73). During innate immune responses, the key role of P2X7R is to activate the assembly of nucleotide-binding domain (NOD) like receptor protein 3 (NLRP3) inflammasome rapidly, which could consecutively facilitate caspase-1 meditated maturation and release of the pro-inflammatory cytokines interleukin-1β and interleukin-18 to participate in both defense and inflammatory responses (74, 75). For adaptive immune responses, eATP signals *via* P2X7R to boost the activation, proliferation, and chemotaxis of immune cells with consequent stimulation of CD8^+^ and CD4^+^ T cell mediated anti-tumor responses (74, 76, 77). The production of pro-inflammatory cytokines, such as interleukin-1β and interleukin-18, are involved in the activation of B and NK cells (78). Additionally, the stimulation of P2X7R inhibits the tissue-specific immunosuppressive potential of regulatory T cells (Tregs) and facilitated their conversion to T helper 17 (Th17) cells during chronic inflammation (79). On the contrary, P2X7R antagonism increases Tregs and reduces clinical and histological graft-versus-host disease in a humanized mouse model (80). Overall, eATP can provide a variety of strategies to enhance the ability to eliminate malignant cells.

### The CD39-CD73-adenosine axis

The human body always keeps a delicate balance between injury and repair to avoid overcorrection. Over time eATP becomes less inflammatory or even anti-inflammatory due to the recruitment of Tregs and induction of ectoenzymes such as CD39 and CD73 (**Figure 4**) (69). As the critical components of the extracellular adenosinergic pathway, CD39 converts eATP and eADP to eAMP, and then CD73 converts eAMP to immunosuppressive adenosine (81). Moreover, another pathway generating adenosine involves participation of extracellular nicotinamide adenine dinucleotide (NAD^+^), CD38, CD203a, and CD73 (82). Like CD39 and CD73, alkaline phosphatase (ALP) and prostatic acid phosphatase (PAP) also can catalyze the conversion of eATP to adenosine (83, 84). Furthermore, the high concentration of intracellular adenosine can be transported outside the cell *via* equilibrative nucleoside transporters (ENTs) and concentrative nucleoside transporters (CNTs) to maintain balance (85).

The levels of extracellular adenosine are regulated by adenosine-converting enzymes such as adenosine kinase (ADK) and adenosine deaminase (ADA). Among them, ADK adds the residue of phosphoric acid to adenosine and converts it into AMP and ADA separates an amino group from adenosine with the formation of inosine (86). However, in the TME, high concentrations of adenosine binding to the corresponding receptors to inhibit the activation and expansion of various immune cells and promote the immune escape of cancers (86). The four known subtypes of adenosine receptors (A1R, A2AR, A2BR, and A3R), all of which are G-protein coupled receptors (GPCRs), have distinct expression patterns and mediate diverse signaling pathways (87). Regarding the respective role of adenosine receptors, it has been demonstrated that among the four subtypes, adenosine binding to A2AR and A2BR causes an increase in intracellular cyclic adenosine monophosphate (cAMP) and consequently the functional inhibition of immune cells, while A1R and A3R activation leads to tumor growth, cell proliferation and survival in some cases (88–90).

### Immunosuppressive adenosine and TME

Adenosine accumulated in the TME is a major cause of immunosuppression (**Figure 4**). As the main force to eliminate malignant cells, the impairment of CD8^+^ T cells function and metabolic fitness are mediated by the A2AR/PKA/mTORC1 pathway as the main axis, due to the persistent high concentration of adenosine (91). Blocking the interaction of receptor with adenosine by a small-molecule A2AR antagonist can increase the recruitment of CD8^+^ T cells into the tumor and broaden the circulating T cell repertoire (92). Similarly, existing studies also indicate that immunosuppressive adenosine can impair the parenchymal CD4^+^ T cell and B cell response and infiltration (93, 94). Although NK cells rarely infiltrate cancers, their presence in tumor biopsies has been shown to positively associate with increased survival (95). As an intrinsic negative regulator of NK-cell maturation and anti-tumor immune responses, A2AR-mediated adenosine signaling can obviously limit tumor-infiltrating NK cells proliferation and activation (96). At the interface between the innate and adaptive immune system, dendritic cells (DCs) play key roles in inflammation and tumor immunity (97). However, adenosine and cAMP signaling can not only prevent DC maturation and development of effector functions but also skew DC differentiation towards a tolerogenic phenotype with defective CD8^+^ T cell priming capacity (98).

Extensive literature shows that eATP-mediated activation of purinergic receptor is necessary for the maturation and release of interleukin-1β by activated macrophages (99). Nevertheless, adenosine generated by eATP likely contributes to the differentiation and recruitment of tumor-associated macrophages (TAMs) which further amplify adenosine-dependent immunosuppression *via* additional ectonucleotidase activity of cancer cells (100). Myeloid-derived suppressor cells (MDSCs) are considered to be an important contributor to the immunosuppressive TME and thus an obstacle for many cancer immunotherapies. The metabolite adenosine plays a vital role in MDSCs mobilization through several mechanisms to inhibit T cell functions and promote cancer progression (101). In addition, elevated adenosine upregulates CD73 on cancer associated fibroblasts (CAFs) *via* A2BR-mediated pathway, thereby inciting the adenosine-A2BR-CD73 feedforward circuitry, which further augments immunosuppression by activating the non-redundant adenosine-A2AR pathway in immune cells to inhibit immune activation (102). For mesenchymal stromal cells (MSCs), the modulation of the adenosine overall promotes a more aggressive phenotype of cancers and more serious immunosuppressive function (103). Recently, Abhishek Tripathi et al. found a strong correlation between CD73, CD39 and A2AR expression, and Treg gene expression signature. Adenosine activates the high-affinity A2AR receptor, which in turn inhibits infiltrating NK cells and cytotoxic T lymphocytes (CTLs) activity and increases Tregs proliferation to further promote immunosuppression (104). Beyond the task of providing an immune-tolerant TME by helping to determine the activity of immune and inflammatory cells, the adenosine system directly regulates cancer growth and metastatic dissemination through specific receptors that are expressed on cancer cells (105).

Overall, in the context of cancer, the accumulation of extracellular adenosine inhibits the normal function of immune effector cells and facilitate the effect of immunosuppressive cells to foster malignant cells proliferation and migration.

## Adenosine signaling in GC

Extracellular release of the central cellular energy metabolite ATP has although evolved as a natural signal for cellular distress, immunogenic cell death (ICD) and the recruitment and activation of immune cells (106). Ectonucleotidases which up-regulated in many types of cancer, such as CD39 and CD73, rapidly metabolize eATP to immunosuppressive adenosine, thereafter exacerbating immunosuppression in the TME (107).

Similar to other malignancies, the expression of CD39 and CD73 is synergistically increased in GC, causing a poor outcome for patients (61, 108). Under the dysfunction of mitochondria, GC cells preferentially utilize both glycolytic and pentose phosphate pathways rather than electron transport chains to desperately generate ATP, classically recognized as the Warburg effect, to provide substrates for adenosine production (109). Importantly, CD73 is also a hypoxia-responsive gene and promotes the Warburg effect of GC dependent on its enzyme activity to further amplifying adenosine signal transduction (110). Immunosuppressive adenosine can enhance the stemness of GC to resist treatment and promote the expression of epithelial-mesenchymal transition-associated genes to stimulate GC cell invasion and metastasis *via* interaction with A2AR and subsequent activation of the PI3K/AKT/mTOR pathway (111, 112). Furthermore, pathway and gene set enrichment analysis of transcriptome data revealed the modulation role of adenosine in RICS/RhoA signaling, which subsequently inhibited phosphorylation of LIMK/cofilin and promoted β-catenin activation to induce metastasis of GC (63).

Long-term accumulation of adenosine in GC helps to establish the immunosuppressive TME and promote tumor development through its interaction with tumor parenchyma and stromal cells (113). For immune cells, tumor-associated Tregs express more CD39 and CD73 in GC tissue. They also can decompose eATP to adenosine and in turn not only induce apoptosis and inhibit the proliferation of CD8^+^ T cells through the A2AR pathway but also prevent the infiltration of effector T cells into the TME (114, 115). Moreover, Hanyuan Liu et al. found that CD73 high expression GC showed a specific microenvironment with more CD8^+^ T cell infiltration *via* recruiting 902 GC patients to examine CD73 expression and immune contexture, but these CD8^+^ T cells displayed a dysfunctional phenotype for anti-tumor immunity (62). As a bypass pathway for adenosine production, restraining the conversion of NAD^+^ to adenosine can improve the function of effector CD8^+^ T cells and induce the apoptosis of GC cells simultaneously (116).

Though lots of systemic and in-depth researches on the role of the CD39-CD73-adenosine axis in diseases have been implemented, such as cardiovascular diseases, autoimmune disease, gut inflammation, and other cancers, immune checkpoint therapy targeting adenosine pathway in GC is still in the early phase (113, 117–119). With the use of small molecule inhibitors and monoclonal antibodies targeting adenosine pathway, an increasing number of clinical trials designed for GC treatment are ongoing, yet few successful experiences have been identified thus far (**Table 2**). Therefore, further exploration is still needed to complement the deficiencies of this immunotherapy method for GC patients.

**Table 2:** The clinical trials of blocking adenosine signaling in patients with advanced solid tumors.

Target	Status	Drug names	Combination	Trial phase	Clinical trial number
CD39	Recruiting	SRF617	Gemcitabine Albumin-Bound Paclitaxel Pembrolizumab	Phase 1	NCT04336098
	Active	TTX-030	Nab-paclitaxel Gemcitabine	Phase 1	NCT04306900
	Recruiting	ES002023	None	Phase 1	NCT05075564
	Not yet recruiting	ES014	None	Phase 1	NCT05381935
	Recruiting	JS019	None	Phase 1	NCT05374226
	Not yet recruiting	PUR001	None	Phase 1	NCT05234853
CD73	Recruiting	IPH5301	Chemotherapy Trastuzumab	Phase 1	NCT05143970
	Recruiting	PT199	Anti-PD-1 monoclonal antibody	Phase 1	NCT05431270
	Recruiting	Sym024	Anti-PD-1 monoclonal antibody	Phase 1	NCT04672434
	Active	LY3475070	Pembrolizumab	Phase 1	NCT04148937
	Not yet recruiting	HLX23	None	Phase 1	NCT04797468
	Recruiting	AK119	Anti-PD-1/CTLA-4 bispecific antibody	Phase 1	NCT04572152
	Recruiting	IBI325	Anti-PD-1 monoclonal antibody	Phase 1	NCT05119998
	Terminated	GS-1423	mFOLFOX6 Regimen	Phase 1	NCT03954704
	Not yet recruiting	JAB-BX102	Anti-PD-1 monoclonal antibody	Phase 2	NCT05174585
	Active	MEDI9447	Anti-PD-L1 monoclonal antibody	Phase 1	NCT02503774
	Recruiting	INCA00186	Anti-PD-1 monoclonal antibody	Phase 1	NCT04989387
	Recruiting	TJ004309	None	Phase 2	NCT05001347
	Completed	BMS-986179	Anti-PD-1 monoclonal antibody	Phase 2	NCT02754141
A2AR	Not yet recruiting	ILB2109	None	Phase 1	NCT05278546
	Recruiting	EOS100850	None	Phase 1	NCT05117177
	Recruiting	M1069	None	Phase 1	NCT05198349
A2BR	Not yet recruiting	TT-4	None	Phase 2	NCT04976660

## Prospects

The considerable heterogeneity and immunosuppressive TME represent major obstacles to accurate diagnosis and effective treatment in GC patients, leading to ineffective immunotherapy (120). For tumor heterogeneity, the molecular classification of GC extends the potential for personalized treatments to benefit each patient and fulfill the concept of precision medicine (121). The development of GC is a complex process displaying polytropic cell and molecular landscape within the TME, which supports tumor growth, metastasis, and recurrence, and function as the soil for gastric tumorigenesis (122). There is increasing evidence that reprogrammed energy metabolism contributes to the development of tumor suppressive immune microenvironment and influences the course of GC (123).

As a common metabolite, immunosuppressive adenosine has been intensively studied in many benign and malignant diseases, nevertheless, few researchers are currently exploring this avenue in GC. Although the efficacy of multiple small-molecule antagonists and antibodies of CD39-CD73-adenosine signaling pathway are being verified in a variety of diseases, deficiencies such as inefficacy and excessive inflammation cannot be ignored. Based on both, further research should mainly focus on the following aspects to obtain better curative effect:

### Develop new drugs targeting adenosine pathway with higher specificity, less side effect and better efficacy.

Adenosine signaling, as one of the key components in regulating normal immune responses, induces immune tolerance to prevent an overreaction with self and the development of autoimmune disease (124). Due to the clinical experience with adenosine pathway inhibitors in oncology is limited, long-term exposure to these drugs and their association with other anti-tumor treatments could potentially lead to the emergence of systemic multiorgan toxicity (125). Therefore, the development of new drugs should also pay attention to its safety.

### Simultaneously target multiple adenosinergic pathway components to acquire synergistic efficacy.

Multiple pathways can contribute to the production of adenosine, some of them by traditional CD39/CD73-dependent mechanisms, others by alternative pathways. In order to disrupt the adenosine production, Nathalie Bonnefoy et al. generated two antibodies, IPH5201 and IPH5301, targeting human membrane-associated and soluble forms of CD39 and CD73, respectively, and efficiently blocking the hydrolysis of immunogenic ATP into immunosuppressive adenosine. Their results suggested that the concomitant blockade of both CD39 and CD73 immunosuppressive enzymes can limit adenosine-mediated T cell inhibition, thereby enhancing anti-tumor immunity (126). Similarly, the simultaneous inhibition CD39 and CD73 cell surface ectonucleotidases by small molecular inhibitors can enhance the mobilization of bone marrow residing stem cells by decreasing the extracellular level of adenosine (127). In addition, co-targeting CD73 and A2AR strategy is also a promising novel therapeutic strategy for future hepatocellular carcinoma management (128). More interestingly, the alternative pathways can compensate the lack of adenosine production when the CD39/CD73/adenosine axis is blocked (129). Hence, a strong rationale exists for combining several inhibitions with the aim of more completely blunting adenosine production and signaling, but no similar research has been conducted on GC. It is worth noting that the combination therapy may improves the treatment outcome but it also carries more side-effect burden.

### Combine adenosinergic pathway inhibitors with other cancer treatments.

Systemic immunosuppression greatly affects the chemotherapeutic anti-tumor effect. CD39 cell-surface expression and activity is increased in patients with acute myeloid leukemia (AML) upon chemotherapy compared with diagnosis, and enrichment in CD39-expressing blasts is a marker of adverse prognosis in the clinic (130). Furthermore, extracellular vesicles from B cells through CD39 and CD73 vesicle-incorporated proteins hydrolyze eATP from chemotherapy-treated tumor cells into adenosine, thus impairing CD8^+^ T cell responses (131). As receptor for adenosine signaling, elevated A2AR expression was also detected in recurrent tumor tissues with induction chemotherapy (132). These phenomena offer a preclinical proof for the administration of adenosine signaling inhibitors in combination with chemotherapy in cancers, possibly including GC. Notably, the addition of HER2-targeted therapies to first-line chemotherapy has improved the OS of patients with HER2-positive GC, and has become the standard-of-care treatment for this group of patients (133). In breast cancer, high levels of CD73 gene expression are associated significantly with poor clinical outcome and promote resistance to HER2 antibody therapy (134). However, whether inhibitors of adenosinergic signaling pathway can be used to increase the efficacy of HER2-targeted therapy in GC needs to be further demonstrated. Various forms of immunotherapy are proving to be effective at restoring T cell-mediated immune responses that can lead to marked and sustained clinical responses, especially ICIs and CAR T-cell therapy. However, the efficacy of various immunotherapies for solid tumor is still mediocre because of immunosuppression in the TME. Hypoxia and cell damage, as common phenomena in solid tumors, are strongly linked to hallmarks of cancers and facilitate the production of immunosuppressive adenosine. The studies revealed that targeted blockade of CD73 can enhance the therapeutic activity of anti-PD-1 and anti-CTLA-4 monoclonal antibodies and may thus potentiate therapeutic strategies targeting ICIs for colorectal cancer, breast cancer, and prostate cancer (126, 135). Previous studies have shown that adenosine generated by tumor cells potently inhibits CAR T-cell responses through activation of A2AR. Therefore, using either A2AR antagonists or genetic targeting of A2AR using short hairpin RNA can profoundly increase CAR T-cell efficacy, particularly when combined with PD-1 blockade (136). In addition, disrupting A2AR gene in human CAR T-cell with CRISPR-Cas9 increased the anti-tumor function and prevented the exhaustion of CAR T-cells (137). Mechanistically, human A2AR-edited CAR T-cells are significantly resistant to adenosine-mediated transcriptional changes, resulting in enhanced production of cytokines including interferon-γ and tumor necrosis factor-α, and increased expression of JAK-STAT signaling pathway associated genes (138). The purpose of combination therapy is to combine separate mechanisms of action that will make malignant cells more sensitive to therapeutic agent and acquire better curative effect, but no similar research has been conducted on GC.

### Promote adenosine metabolism to attenuate the immunosuppressive ability of TME

In addition to the above methods, accelerating the metabolism of adenosine within TME also can restore an anti-tumor immune competence. Emanuele Sasso et al. encoded adenosine deaminase (ADA)into an oncolytic targeted herpes virus to improve enzyme secretion for the metabolism of adenosine, and the clearance of adenosine within the TME reversed HER2-positive breast cancer resistance to trastuzumab (139).

## Conclusion

The growth and progression of solid tumors are strongly affected by adenosine metabolic changes and interplay with the TME that sustain tumor development and immune escape. We explored the expression pattern and prognostic value of common immune checkpoints in GC patients *via* GEPIA database. Compared with other targets, adenosinergic pathway plays an indispensable role in the occurrence and development of GC, especially CD73. The components of adenosinergic pathway on both GC cells and immune cells sustains immunosuppressive TME by affecting multiple aspects of the immune response. Furthermore, some emerging antagonists of adenosinergic pathway show therapeutic potential in the preliminary studies of other malignancies. Therefore, these findings uncovered a mechanism by which immunosuppressive adenosine participates in the immune tolerance of GC, implying the potential of adenosinergic pathway as a therapeutic target or predictive marker for GC patients. However, On the basis of the limited evidence available as of now, elaborate clinical evaluation is further warranted to confirm whether the adenosinergic-targeting therapies are suitable for GC patients.

## Author contributions

J-QW: writing of original manuscript. L-YD: revision of the manuscript. X-JC: language modification of the manuscript. All authors contributed to the article and approved the submitted version.

## Funding

This work was supported by Wenzhou Science & Technology Bureau Foundation (Grant No. Y2020144 to X-JC) and National Natural Science Foundation of China under (Grant No. 81902151 to L-YD).

## Acknowledgments

All authors contributed to the conception of the study and the preparation and approval of the paper.

## Conflict of interest

The authors declare that the research was conducted in the absence of any commercial or financial relationships that could be construed as a potential conflict of interest.

## Publisher’s note

All claims expressed in this article are solely those of the authors and do not necessarily represent those of their affiliated organizations, or those of the publisher, the editors and the reviewers. Any product that may be evaluated in this article, or claim that may be made by its manufacturer, is not guaranteed or endorsed by the publisher.

## Abbreviations

GC, gastric cancer; ICI, immune checkpoint inhibitor; AGC, advanced gastric cancer; GEPIA, Gene Expression Profiling Interactive Analysis; EGC, early gastric cancer; HER2, human epidermal growth factor receptor 2; CAR, T-cell chimeric antigen receptor-modified T cell; CTLA-4, cytotoxic T lymphocyte-associated antigen 4; PD-1/PD-L1, programmed cell death receptor 1/programmed cell death ligand 1; OS, overall survival; PFS, progression-free survival; eAMP, extracellular adenosine monophosphate; eADP, extracellular adenosine diphosphate; eATP, extracellular adenosine triphosphate; TME, tumor microenvironment; NK, natural killer cell; Tregs, regulatory T cells; DC, dendritic cell.
